# The NMDA Receptor Subunit (GluN1 and GluN2A) Modulation Following Different Conditions of Cocaine Abstinence in Rat Brain Structures

**DOI:** 10.1007/s12640-021-00350-0

**Published:** 2021-03-24

**Authors:** Irena Smaga, Karolina Wydra, Agata Suder, Małgorzata Frankowska, Marek Sanak, Lucia Caffino, Fabio Fumagalli, Małgorzata Filip

**Affiliations:** 1 Department of Drug Addiction Pharmacology, Maj Institute of Pharmacology Polish Academy of Sciences, Smętna 12, 31-343 Kraków, PL Poland; 2 Department of Internal Medicine, Jagiellonian University Medical College, Skawińska 8, 31-066 Kraków, PL Poland; 3 Department of Pharmacological and Biomolecular Sciences, Università Degli Studi Di Milano, Via Balzaretti 9, 20133 Milano, Italy

**Keywords:** Cocaine abstinence, Cocaine self-administration, NMDA receptor subunit

## Abstract

Different neuronal alterations within glutamatergic system seem to be crucial for developing of cocaine-seeking behavior. Cocaine exposure provokes a modulation of the NMDA receptor subunit expression in rodents, which probably contributes to cocaine-induced behavioral alterations. The aim of this study was to examine the composition of the NMDA receptor subunits in the brain structures in rats with the history of cocaine self-administration after cocaine abstinence (i) in an enriched environment, (ii) in an isolated condition, (iii) with extinction training, or (iv) without instrumental task, as well as the *Grin1* (encoding GluN1) and *Grin2A* (encoding GluN2A) gene expression were evaluated after 10-day extinction training in rat brain structures. In the present study, we observed changes only following cocaine abstinence with extinction training, when the increased GluN2A subunit levels were seen in the postsynaptic density fraction but not in the whole homogenate of the prelimbic cortex (PLC) and dorsal hippocampus (dHIP) in rats previously self-administered cocaine. At the same time, extinction training did not change the *Grin1* and *Grin2A* gene expression in these structures. In conclusion, NMDA receptor subunit modulation observed following cocaine abstinence with extinction training may represent a potential target in cocaine-seeking behavior.

**Supplementary Information:**

The online version contains supplementary material available at 10.1007/s12640-021-00350-0.

## Introduction

Cocaine exposure causes structural and functional adaptations within the neural reward circuitry, which seem to be at the core of cocaine use disorder. These neurobiological adaptations in the brain promote craving for cocaine, while dysfunction of reward motivation induces frequent drug-taking. Over the past decade, most research has suggested that the development of substance use disorder is related with cocaine-induced plasticity in the glutamatergic transmission (Bellone and Lüscher 2012; Kalivas 2004). Different neuronal alterations within glutamate signaling (glutamate levels, receptors, and transporters) may be involved in the development of drug craving by enhancing the incentive motivational value of cocaine (Kalivas 2004). In particular, changes in the NMDA receptor subunit composition may represent a potential cellular mechanism leading to cocaine-seeking behavior.

NMDA receptors play a significant role in several physiological processes, and their modulation is involved in the pathogenesis of different brain disorders including substance use disorders (Smaga et al. 2019, 2021). NMDA receptors are tetrameric protein complexes composed of two obligatory GluN1 subunits and two GluN2 (A-D) subunits. NMDA receptors require the membrane depolarization and binding of both endogenous glutamate via the GluN2 subunit and the coagonist glycine via the GluN1 subunit, which results in the opening of channel pores permeable to several ions (Traynelis et al. 2010).

The transition from cocaine abuse to dependence, as well as the transition from cocaine dependence to cocaine abstinence, may involve changes in the NMDA receptor subunit composition (Smaga et al. 2019). Following contingent-drug delivery in drug self-administration paradigm, animals typically undergo drug-forced abstinence under either extinction training or withdrawal conditions. Withdrawal usually occurs outside the experimental chambers (in a home cage or in an enriched environment), while extinction training in the experimental chambers produces reduction (extinction) of the behavioral response (e.g., decreases in active lever pressing that no longer results in drug delivery) (Bossert et al. 2013). Little is known about the changes within NMDA receptor subunit composition in different condition of drug-free period. Recently, we have shown the increased levels of GluN1 and GluN2A subunit expression in the nucleus accumbens after the last cocaine self-administration session (Smaga et al. 2020). Increased accumbal GluN1 levels were maintained after 10-day cocaine abstinence with extinction training, while cocaine abstinence in other conditions (isolation, enriched environment, experimental cage without instrumental task) normalized the GluN1 levels observed in rats previously self-administering cocaine (Smaga et al. 2020). Therefore, NMDA receptors and the composition of the receptor subunit are of major interest in their role in the cocaine use disorder, since the role of the NMDA receptor subunit seems to be critical in drug-free periods in different conditions of abstinence.

The aim of this study was to investigate the composition of the NMDA receptor subunits- focusing our attention on GluN1 and GluN2A subunits- in abstinent rats following a history of cocaine self-administration. In particular, we evaluated their expression in the total homogenate and post-synaptic density (PSD) fraction of the dorsal (dHIP) and ventral (vHIP) hippocampus, dorsal (dSTR) and ventral (vSTR) striatum, infralimbic (ILC) and prelimbic (PLC) cortex, and basolateral amygdala (BLA). Furthermore, the *Grin1* (encoding GluN1 subunit) and *Grin2A* (encoding GluN2A subunit) gene expressions were determined using microarray analysis to evaluate changes in the rat brain structures after cocaine abstinence with extinction training. Cocaine-forced abstinence was performed in an enriched environment (in big home cages with social influence and toys without any influence of cocaine or the drug-associated conditioned stimulus), in an isolated condition (home cages without any influence of cocaine or drug-associated conditioned stimulus), under extinction training (daily sessions with no delivery of cocaine nor the presentation of the conditioned stimulus − tone + light associated with cocaine delivery), or exposure to experimental chambers without access to accomplish instrumental response (levers removed).

## Materials and Methods

### Animals

**Fig. 1:**
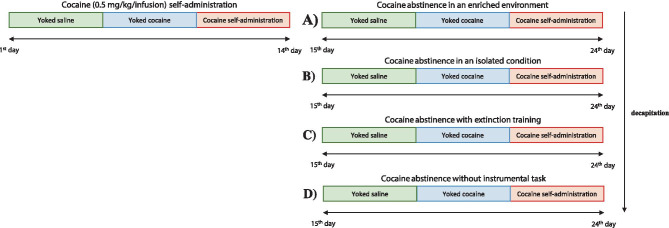
Diagram illustrating the experimental procedure. After the 14th self-administration session, rats were separated to undergo 10 days of cocaine abstinence in four housing conditions: **a** cocaine abstinence in an enriched environment; **b** cocaine abstinence in an isolated condition; **c** cocaine abstinence with extinction training; and **d** cocaine abstinence without the instrumental task

Male Wistar rats (225–250 g; Charles River, Sulzfeld, Germany) were housed in collective cages in a room maintained at 22 ± 2°C and 55 ± 10% humidity under a 12-h light–dark cycle (between 6.00 a.m. and 6.00 p.m.). Rats have free access to water and standard animal food (VRF1 pellets, UK) except for the initial training to lever presses and the first 3 days of self-administration procedure. All the experiment procedures were carried out in accordance with EU directive 2010/63/EU and with approval of the Local Ethics Commission at the Maj Institute of Pharmacology Polish Academy of Sciences in Krakow, Poland (1235/2015). The experimental protocol steps are presented in Fig. 1.

### Drugs

Cocaine hydrochloride (Toronto Research Chemicals, Canada) was dissolved in sterile 0.9% NaCl and given intravenously in a volume of 0.1 ml per infusion.

### Intravenous Catheter Implantation

Rats were anesthetized (ketamine hydrochloride, 75 mg/kg, i.m. and xylazine, 5 mg/kg, i.m.; Biowet, Poland) and implanted with a silastic catheter in the external right jugular vein, as described previously (Bystrowska et al. 2014). Meloxicam (Metacam, Boehringer IIngelheim; 5 mg/kg, s.c.) was used to reduce post-operative pain during 3 days after surgery. Catheters were flushed daily with 0.2 ml of saline solution containing cefazolin (100 mg/ml; Biochemie GmbH, Austria) and heparin (100 U/ml; Biochemie GmbH, Austria) to prevent catheter non-patency as a result of blood clotting during the recovery period (7 days).

### Initial Training

Rats were trained to press lever for food pellets (VRF1 pellets, UK) under a fixed ratio (FR) from 1 to 5 reinforcement schedule from 2 to 3 days for 2 h daily in a sound attenuated, standard operant conditioning chambers (Med-Associates, St. Albans, VT, USA). Starting 24 h prior to the food training session, rats received rations of ~ 20 g of chow daily. Each chamber was equipped with reward feeder presses on active lever resulted in the delivery of 0.1 ml of sweetened milk and continued until rats reached a criterion of 100 active lever presses, while the inactive lever was not programmed.

### Cocaine Self-administration

Following food training period, rats began the self-administration procedures using the same standard operant chambers. During cocaine self-administration (0.5 mg/kg/infusion, 2 h/day, 14 days), rats obtained a minimum of 10 infusions per day. Active lever presses during cocaine self-administration resulted in delivery dose of cocaine, as well as the stimulus light illumination (24-V) above the active lever and a tone presentation (2000 Hz; 15 dB), simultaneously for a programmed duration of 5 s. A 20-s timeout followed the delivery of each infusion during which time active lever presses were recorded but had no consequences. Presses on the inactive lever were recorded, but not reinforced. Acquisition of the conditioned operant response lasted until subjects met a stable average of three consecutive days (a standard deviation within those days of < 10% of the average) (Wydra et al. 2015).

Rats were tested simultaneously in groups with rats serving as yoked controls that received an injection of saline (yoked saline) or cocaine (yoked cocaine), which was not contingent on the response, and each time, a response-contingent injection of 0.5 mg/kg cocaine was self-administered by the paired rat. Unlike self-administering rats, lever pressing by the yoked rats was recorded but had no programmed consequence. After the 14th (2-h) self-administration session, all animals, which met the maintenance criterion, were separated to undergo 10 days of cocaine abstinence in four housing conditions: (i) cocaine abstinence in an enriched environment, (ii) cocaine abstinence in an isolated condition, (iii) cocaine abstinence with extinction training, and (iv) cocaine abstinence without the instrumental task.

### Yoked Self-administration Procedure

To distinguish the pharmacological effects of cocaine from those related to motivation, we used the yoked procedure. Briefly, the yoked cocaine rats received an infusion of cocaine at the same dose and rate as the self-administration group, while yoked saline rats received an infusion of saline. The levers pressed by the yoked rats were recorded but had no programmed consequences.

### Cocaine Abstinence Procedures

#### Cocaine Abstinence in an Enriched Environment

During abstinence in an enriched environment, rats (*N* = 8 rats/group) lived in standard large cages that housed four animals. These rats were handled several times per day in cages that contained bedding, two water bottles, short or long PVC pipes, pieces of cotton material mounted to the top of the cage, and small plastic and/or wood toys. Toys, cotton material, and PVC pipes were changed 3 times per week to maintain novelty.

#### Cocaine Abstinence in an Isolated Condition

During abstinence, animals (*N* = 7 rats/group) lived individually in plastic cage with white walls (isolation cage) in a room to which only the experimenter had access to reduce social interactions. Animals were handled once per week.

#### Cocaine Abstinence with Extinction Training

Rats (*N* = 8 rats/group) following the last cocaine self-administration session underwent an extinction training. During the 2-h daily extinction training sessions, all animals had no delivery of cocaine or the presentation of the conditioned stimulus. Animals that met the extinction criterion (i.e., responses on the active lever fell to < 10% of the responses at the active lever reached during self-administration of cocaine) were sacrificed immediately following the last (10th) session of extinction training for Western blot analyses. Separated three groups of animals (*N* = 8 rats/group) were sacrificed following the last (10th) session of extinction training for microarray analyses; these animals were the same as in our previous study (Sadakierska-Chudy et al. 2017).

#### Cocaine Abstinence Without the Instrumental Task

Animals (*N* = 8 rats/group) following the last cocaine self-administration session underwent a 10-day training in self-administered operant chambers, where the rats had no presentation of the conditioned stimulus and levers (only home light) during 2-h daily sessions.

### Brain Structures Isolation

All animals were decapitated in the 10th cocaine abstinence day. Rat brains were rapidly removed on ice-chilled surface. Selected brain structures (i.e., ILC, PLC, dHIP, vHIP, dSTR, vSTR, BLA) were isolated according to The Rat Brain Atlas (Paxinos and Watson 1998), immediately frozen on dry ice, and stored at −80°C for Western blot analyses. Separated groups of animals were decapitated following the last (10th) session of extinction training (these animals were the same as in our previous study (Sadakierska-Chudy et al. 2017)), and prefrontal cortex, dSTR, and HIP were isolated according to The Rat Brain Atlas (Paxinos and Watson 1998), immediately frozen on dry ice, and stored at −80°C for microarray analyses.

### Molecular Biology Analyses

#### Western Blot

Brain structures were homogenized in a Teflon-glass potter in a cold buffer (0.32 M sucrose buffer pH 7.4 containing 1 mM HEPES, 1 mM MgCl_2_, 1 mM NaHCO_3_, and 0.1 mM PMSF, a cocktail of protease, and phosphatase inhibitors). The homogenate was centrifuged at 1000×*g* for 10 min obtaining a pellet (P1, the nuclear fraction). The supernatant (S1) was then centrifuged at 9000×*g* for 15 min to obtain a fraction S2, cytosolic proteins and a pellet P2, a crude membrane fraction. Then, P2 was resuspended in 1 mM HEPES with protease and phosphatase inhibitors and centrifuged at 100,000×*g* for 1 h. The pellet (P3) was resuspended in a buffer (75 mM KCl and 1% Triton X-100) in a glass-glass potter and centrifuged at 100,000×*g* for 1 h obtaining supernatant (S4, Triton X-100 soluble fraction) and the pellet (P4, PSD, or Triton X-100 insoluble fraction (TIF)). The pellet (P4) was homogenized in a glass–glass potter in 20 mM HEPES with protease and phosphatase inhibitors and stored at −20°C at the presence of glycerol 30%. For protein determination, a bicinchoninic acid assay (BCA) protein assay kit (Serva, Germany) was used.

Homogenate (10 μg of proteins) and PSD fraction (5 μg of proteins) were then denatured in SDS-PAGE sample buffer (62.5 mM Tris-HCl, pH 6.8, 10% glycerol, 2% SDS, and 0.001% bromophenol blue) containing 5% β-mercaptoethanol for 2 min at 85°C, next chilled 2 min in ice, heated 5 min at 85°C, and finally chilled 2 min in ice. Protein samples were resolved by electrophoresis in 4–15% gradient precast polyacrylamide gels (Bio-Rad, Poland) and transferred to a polyvinylidene difluoride (PVDF) membrane. Membranes were blocked in 3% non-fat dry milk, and separate sets of membranes were probed with mouse anti-GluN1 monoclonal antibody (1:1000; 32-0500, Thermo Fisher Scientific, USA) and rabbit anti-GluN2A polyclonal antibody (1:1000; A-6473; Molecular Probes, The Netherlands). The expression of NMDA receptor subunits was evaluated relative to that of β-actin control protein using mouse monoclonal antibody at dilution of 1:1000 (A5441; Sigma-Aldrich, USA). Blots were washed and incubated with goat anti-rabbit secondary antibody (1:6000; 926-68071; Li-cor, USA) or goat anti-mouse (1:6000; 926-32210; Li-cor, USA) and visualized with a fluorescence detection Odyssey Clx (Li-cor, USA). Analysis was performed using Image Studio v.2.1. All data were expressed as % of control.

#### RNA Isolation

The isolation of DNA and RNA was performed using the RNA/DNA/PROTEIN Purification Plus Kit (Norgen Biotek, Canada). Briefly, the brain structures (prefrontal cortex, HIP, and dSTR) from rats that underwent cocaine abstinence with extinction training were homogenized (30 s at 3000 rpm, then 2 × 30 s at 2500 rpm; Bioprep-24 Homogenizer (Aosheng, China)) with ceramic beads and lysis buffer. RNA samples were eluted in nuclease-free water preheated to 60°C and purified from DNA (RNA Clean-Up kit; Syngen, Poland). The quantity and quality of the isolated RNA samples were determined using a NanoDrop ND-1000 Spectrophotometer (Thermo Scientific, USA) and agarose gel electrophoresis, and the RNA integrity was checked using chip-based capillary electrophoresis with an RNA 6000 Nano Chip Kit and an Agilent Bioanalyzer (Agilent Technologies, USA).

#### Microarray Analysis

*Grin1* and *Grin2A* gene expression was performed using the Rat 4 × 44 K Gene Expression Array v2 (Agilent Technologies, USA) in rat brain structures. Sample labeling and hybridization were performed according to the Agilent One-Color Microarray-Based Gene Expression Analysis protocol. Four pools of RNA per group (RNA from two rats at equal concentrations; 2 μg of total RNA) were converted to complementary DNA (cDNA) and transcribed into complementary RNA (cRNA) in the presence of cyanine 3-UTP. Then, the labeled cRNAs (1 μg) were fragmented and hybridized to the array for 17 h at 65°C with rotation and washed to remove nonspecific hybridization. The Agilent Microarray Scanner and Feature Extraction software (v 11.0.1.1) (Agilent Technologies, USA) was used to image acquisition and feature extraction for the array. Subsequent quantile normalization and data processing were carried out using the GeneSpring GX software, v. 12.1 (Agilent Technologies, USA).

### Statistical Analyses

All data were expressed as the mean ± SEM. In behavioral experiments, the number of responses on the “active” and “inactive” lever and number of infusions were analyzed using one- or multi-way analysis of variance (ANOVAs) for repeated measurements, the latter analysis followed by post hoc Newman-Keuls test. In neurochemical studies, statistical analyses were performed with one-way ANOVA, followed by Dunnett’s test to analyze differences between group means. One-way ANOVA followed by a Bonferroni post hoc test for gene expression data was used. *P* < 0.05 was considered statistically significant.

## Results

### Behavioral Effects

#### Cocaine Self-administration

**Fig. 2:**
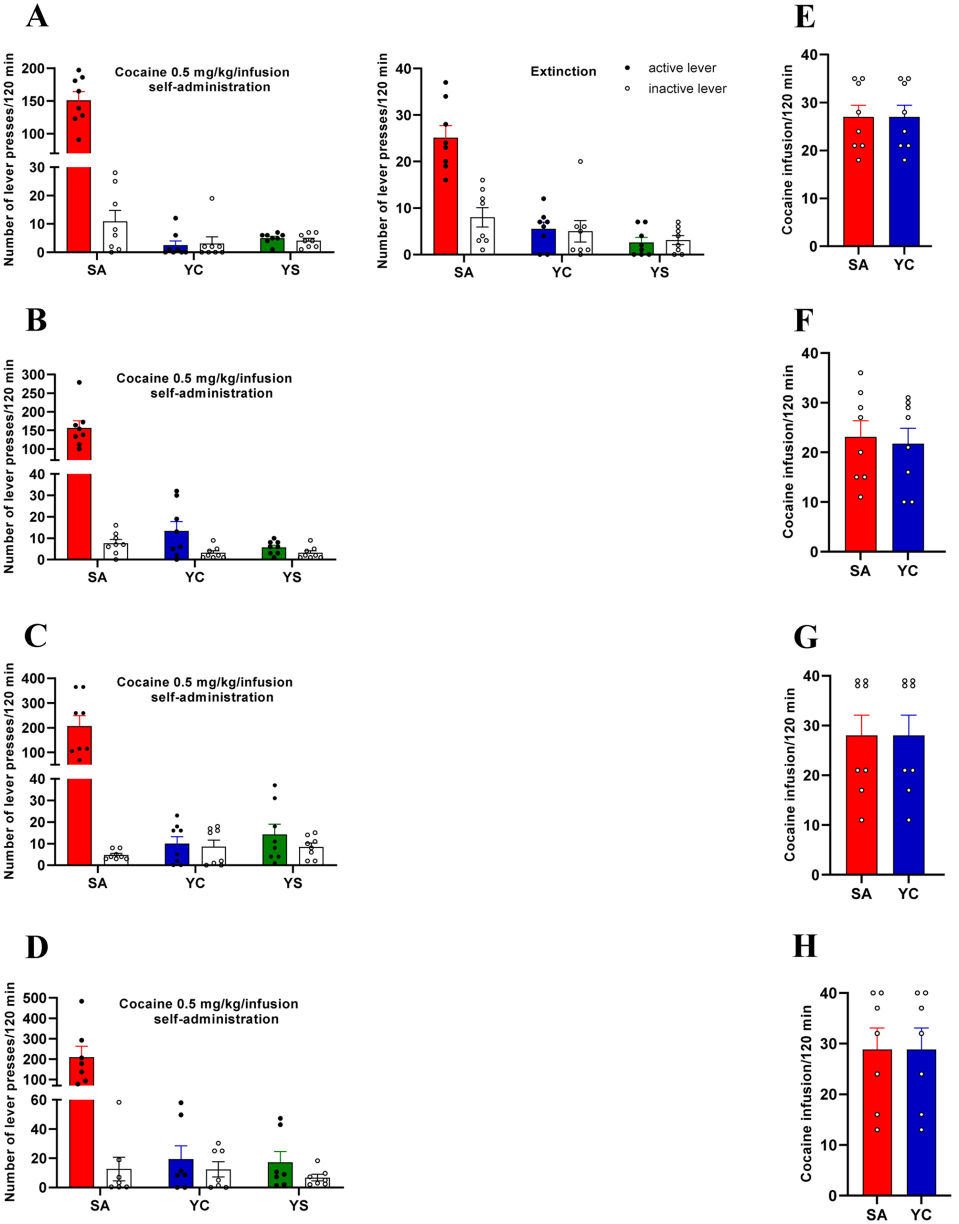
The number of lever presses in cocaine self-administering rats: **a** cocaine abstinence with extinction training (*n* = 8 rats/group); **b** cocaine abstinence without the instrumental task (*n* = 8 rats/group); **c** cocaine abstinence in an enriched environment (*n* = 8 rats/group); **d** cocaine abstinence in an isolated condition (*n* = 7 rats/group); and the number of cocaine infusions during cocaine self-administration (0.5 mg/kg/infusions); **e** cocaine abstinence with extinction training; **f** cocaine abstinence without the instrumental task. **g** Cocaine abstinence in an enriched environment; **h** cocaine abstinence in an isolated condition. Data are presented as the mean ± SEM for 7–8 rats/group

All rats acquired cocaine self-administration (i.e., they received > 23 infusion/2 h under 0.5 mg/kg/infusion) and displayed < 10% variation in the number of cocaine infusions in 14 daily sessions rats. The mean number of cocaine infusions per day during the last 3 self-administration days varied from 22 to 28. The mean of total cocaine intake during 14 days for four cocaine self-administered groups ranged from 157 ± 7 to 186 ± 24 mg/rat. Animals, divided in 4 groups, underwent cocaine abstinence in an enriched environment, in an isolated condition, extinction training, or abstinence in the experimental cage without the instrumental task. During the 3 last cocaine self-administration sessions, behavioral responding in four analyzed groups was stable (day × group × lever (*F*(6, 108) = 0.827; *p* = 0.551, groups *F*(3, 54) = 0.788; *p* = 0.505), readily discriminated between the inactive and active lever *F*(1, 54) = 96.74; *p* < 0.001. Similarly, daily cocaine intake between four groups self-administering cocaine did not differ (day × groups *F*(6, 54) = 0.973, *p* = 0.452). During the 3 last training sessions, the responding in the yoked cocaine (day × group × lever *F*(6, 108) = 1.187; *p* = 0.318) and the yoked saline (day × group × lever *F*(6, 108) = 1.405; *p* = 0.218) was comparable in four analyzed groups (Fig. 2).

### Molecular Biology Analyses

#### Expression of GluN1 Subunit

Cocaine-forced abstinence did not produce changes in the GluN1 expression levels in rats after 10-day cocaine abstinence in different conditions (Table S1, the supplementary material).

#### Expression of GluN2A Subunit

**Fig. 3:**
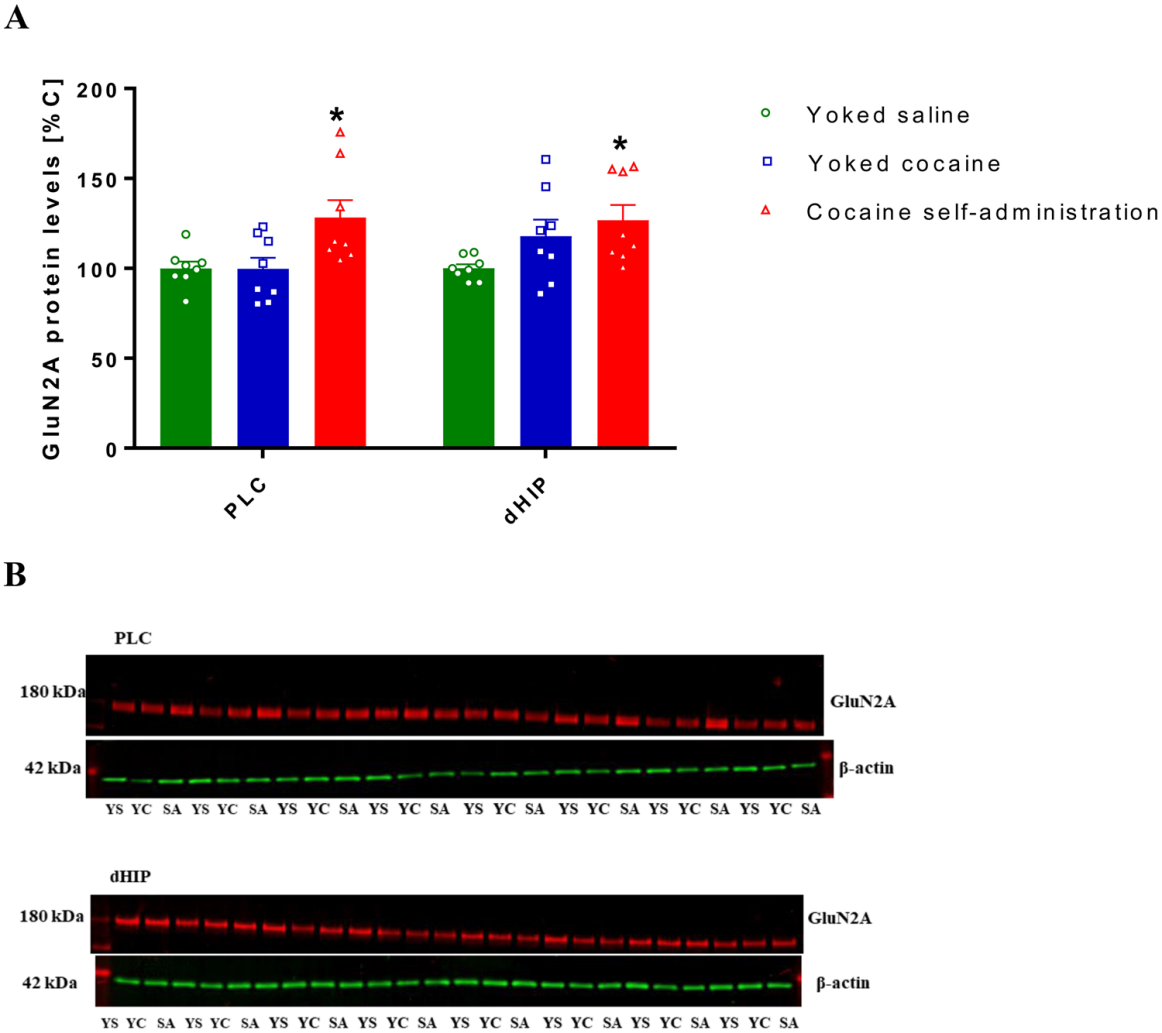
Changes in the expression of GluN2A subunit in the PSD fraction of PLC and dHIP in rats following cocaine abstinence with extinction training **a**. Full membranes for GluN2A subunit are presented **b**. PLC-prelimbic cortex, dHIP-dorsal hippocampus, PSD-postsynaptic density, YS-yoked saline, YC-yoked cocaine, SA-cocaine self-administration. All data are expressed as mean ± SEM. *N* = 8 rats/group. *p < 0.05 vs. yoked saline

Cocaine-forced abstinence did not produce changes in the GluN2A expression levels in rats housed in an enriched environment (Fig. S1a; S2a, the supplementary material) and in an isolated condition (Fig. S1b; S2b, the supplementary material) previously self-administering cocaine, as well as in rats following abstinence without the instrumental task (Fig. S1d; S2d, the supplementary material). Ten days of extinction training increased the expression of the GluN2A subunit only in the PSD fraction of PLC (*F*(2, 21) = 5.517; *p* = 0.012) and dHIP (*F*(2, 21) = 3.507; *p* = 0.048) (Fig. 3), but the expression of this subunit did not change in other structures (Fig. S2c, the supplementary material) of rats with a previous history of cocaine self-administration. At the same time, the expression of the GluN2A subunit did not change in the whole homogenate (Fig. S1c, the supplementary material).

#### Gene Expression

**Fig. 4:**
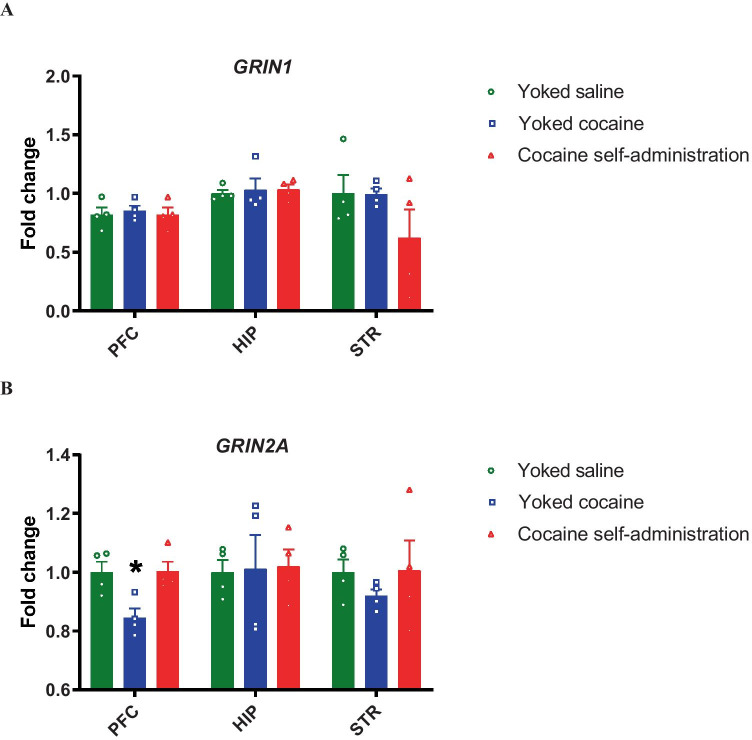
Fold changes regarding *Grin1* and *Grin2A* gene expression following cocaine abstinence with extinction training in the rat brain structures. PFC-prefrontal cortex, HIP-hippocampus, STR-striatum. The data are presented as means ± SEM, *n* = 4 pools of RNA/per group. *p < 0.05 vs. cocaine self-administration group

The *Grin1* and *Grin2A* gene expression did not change after 10 days of extinction training in the rat brain structures in rats previously self-administering cocaine vs. yoked saline and yoked cocaine group (Fig. 4). A decrease in *Grin2A* gene expression was shown in the prefrontal cortex in rats passively administered cocaine (yoked cocaine) vs. cocaine self-administration group (*F*(2, 9) = 7.28; *p* = 0.013) (Fig. 4).

## Discussion

In the present study, we examined the expression of the NMDA receptor subunits in selected rat brain structures during different conditions of cocaine abstinence. We show that 10 days of cocaine abstinence with extinction training evoked an increase in the GluN2A subunit levels in the PSD fraction of the PLC and dHIP in rats previously self-administered cocaine, without any effect on the *Grin2A* gene expression.

The level of GluN2A subunit increased in the dHIP after cocaine abstinence with extinction training in animals previously self-administering cocaine only in the PSD fraction, but not in the whole homogenate. Additionally, it should be noted that the expression of gene *Grin2A* encoding the GluN2A subunit did not change in rats following the 10-day drug-free period, which suggests that the increased expression of this subunit in the PSD fraction may indicate an increased trafficking of these subunits into the synapse surface rather than increased synthesis of GluN2A. Higher level of GluN2A subunit expression in the dHIP is characteristic only for the extinction training. This observation is in line with the evidence that dHIP plays a principal role in the regulation of the reconsolidation of contextual cocaine memories that direct instrumental cocaine-seeking behavior (Ramirez et al. 2009). Src family of tyrosine kinase (SFK)-dependent phosphorylation of GluN2A subunits promotes synaptic strengthening and LTP (Yang et al. 2012), and it is necessary for context-elicited cocaine-seeking behaviors (Xie et al. 2013). Additionally, GluN2A-containing NMDA receptors induce Ras-GRF2-dependent LTP in hippocampal neurons (Lemay-Clermont et al. 2011). An increase in the functional GluN2A subunit level in the dHIP seems to be obligatory during memory reconsolidation. In fact, injection into dHIP PP2 (an ATP-competitive inhibitor of SFKs) administered following exposure to the cocaine-paired context, but not the home cage, reduced the GluN2A subunit activation, as well as the subsequent cocaine-seeking behavior (Wells et al. 2016). Moreover, the injection of NVP-AAM077, a GluN2A subunit antagonist, directly into dHIP following or in the absence of cocaine-memory reactivation attenuated subsequent drug context-induced cocaine-seeking behavior in a memory reactivation-dependent manner (Wells et al. 2016). Extinction training reduces drug-seeking behavior to the drug-associated conditioned response by extinguishing contingency between drug seeking and delivery of the drug reward. It is believed that extinction training is not simply the removal of a previously formed association, but it involves the generation of a new memory that competes with the initial memory for control of behavior (Hutton-Bedbrook and McNally 2013; Torregrossa and Taylor 2013). In fact, after extinction training: (i) drug-seeking behavior can be reactivated, (ii) the retraining of self-administration after extinction is considerably less compared with original training, (iii) drug-seeking resumes after lengthy periods of extinction training indicating that the original drug memory remains, (iv) extinction is context-specific, which means that original memory of drug reinforcement is kept (Bossert et al. 2013; Hutton-Bedbrook and McNally 2013). These findings are supported by the study in which increased levels of GluN2A in the HIP was seen after 10-day extinction training in rats previously self-administered cocaine (Pomierny-Chamiolo et al. 2015); however, such increase was not observed immediately after cocaine self-administration session (Pomierny-Chamiolo et al. 2015).

Interestingly, changes in the GluN2A subunit levels were observed in the PLC in the PSD fraction, where an increase in this protein level was reported in rats previously administered cocaine after 10 days of extinction training. Neither GluN2A in the whole homogenate nor the *Grin2A* expression changed in this structure, suggesting the trafficking of these subunits into the synapse surface. In postsynaptic densities, NMDA receptors are structurally organized in a large macromolecular signaling complex consisting of scaffolding/adaptor proteins (e.g., PSD95, PSD93, and SAP102) that link these receptors to the cellular cytoskeleton (Smaga et al. 2019). The scaffolding proteins primarily serve as a receptor anchor; however, recent studies have demonstrated its role in the regulation of intracellular signaling and internalization. Through their many protein-interacting domains, the PSD proteins are able to regulate directly and/or indirectly the dynamics of postsynaptic receptors, thereby impacting neuroplasticity as glutamatergic neurotransmission takes place primarily at the postsynaptic densities. An increase of the GluN2A subunit has been also reported previously in the whole homogenate of the prefrontal cortex after 10-day extinction training in rats previously self-administering cocaine and passively administered cocaine (Pomierny-Chamiolo et al. 2015). The reasons for these differences between the present and Pomierny-Chamioło et al. (2015) results are probably related to specific brain region examined; in fact, in the present study, a small part of the prefrontal cortex- PLC -was evaluated, while in the study of Pomierny-Chamioło et al. (2015), the whole prefrontal cortex was investigated. It should be noted that an increase in the GluN2A subunit level was not observed immediately after the last cocaine self-administration session (Pomierny-Chamiolo et al. 2015), but this increase was associated with the drug-free period. PLC mediates the action-outcome learning, while ILC is responsible for stimulus-response, which are two forms of learning to control over instrumental responding (Kirschmann et al. 2019). The glutamatergic activity in the PLC afferents to the nucleus accumbens core is necessary to induce reinstatement by cocaine or cues (Stefanik et al. 2013). Pharmacological inactivation of PLC blocked cocaine-induced reinstatement of active lever pressing (Shen et al. 2014). It was shown that cocaine self-administration reduced the phospho-GluN2A levels in the PLC (Go et al. 2016) probably by the activation of striatal-enriched tyrosine phosphatase (STEP) (Sun et al. 2013). Accordingly, increased GluN2A level in the PLC seems to be a compensatory mechanism that occurs after 10-day extinction training. Furthermore, infusion of the GluN2A-containing NMDA receptor antagonist (3-chloro-4-fluoro-N-[4-[[2-(phenylcarbonyl)hydrazino]carbonyl]benzyl] benzenesulfonamide) (TCN-01) into the PLC inhibited the BDNF (brain-derived neurotrophic factor)-mediated increase in phospho-GluN2A (Go et al. 2016). Similarly, PP2, the SFK inhibitor administered during the last session of cocaine self-administration into the PLC prior to BDNF infusion, also blocked the phosphorylation of the NMDA receptor subunit mediated by BDNF, as well as attenuated suppressive effect of BDNF on cue-induced reinstatement in rats previously self-administered cocaine (Barry and McGinty 2017).

Neither cocaine abstinence in an enriched environment, nor in an isolated condition, nor abstinence without the instrumental task change the composition of the NMDA receptor subunit. In line with these observations, we have recently shown increased accumbal levels of the GluN1 subunit in rats following a drug-free period with extinction training previously self-administering cocaine, while other conditions of abstinence abolished the higher levels of GluN1 and GluN2A observed after cocaine self-administration in the nucleus accumbens (Smaga et al. 2020). It was suggested that environmental conditions may have a critical role in cocaine use disorder. In fact, the isolation increased the risk of relapse, while enriched environment and behavioral cue-extinction therapy reduced cocaine-seeking behavior (Goeders 2003; Solinas et al. 2009). Unfortunately, treatments based on manipulations of learning and memory processes involved in encoding the associations non-reinforced exposure to drug-related stimuli or the drugs themselves have produced disappointing results in human addicts (Bossert et al. 2013).

## Conclusions

Our results showed that different conditions of cocaine abstinence did not produce changes in the GluN1 and GluN2A subunit protein expression, except cocaine abstinence with extinction training. Ten-day drug-free period with extinction training procedure eliminated the cocaine injections and cue-contingent presentations and provoked reduction in the active lever pressing. This state was associated with higher level of the GluN2A subunit levels in the PSD fraction of the PLC and dHIP in rats previously self-administering cocaine, without any effect on *Grin2A* gene expression, which suggest that the latter changes were related with cellular trafficking of these subunits. We conclude that the NMDA receptor subunit modulation observed following cocaine abstinence with extinction training may represent a potential target in cocaine-seeking behavior.

## Supplementary Information

Below is the link to the electronic supplementary material.

Supplementary file1 (DOCX 608 KB)

## Abbreviations

BDNF: Brain-derived neurotrophic factor
dHIP: Dorsal hippocampus
dSTR: Dorsal striatum
ILC: Infralimbic cortex
LTD: Long-term depression
LTP: Long-term potentiation
NMDA: N-methyl-D-aspartate
PLC: Prelimbic cortex
vHIP: Ventral hippocampus
vSTR: Ventral striatum
