# Supplementary material for: The NMDA Receptor Subunit (GluN1 and GluN2A) Modulation Following Different Conditions of Cocaine Abstinence in Rat Brain Structures
Source: Neurotox Res. 2021 Mar 24;39(3):556–65. doi: 10.1007/s12640-021-00350-0 (PMC8096759; doi:10.1007/s12640-021-00350-0)
Supplement: Supplementary file 1 — Supplementary file1 (DOCX 608 KB) [file 12640_2021_350_MOESM1_ESM.docx]

**Supplementary Materials**

**Table S1.** Changes in the expression of GluN1 subunit in the whole homogenate (H) and in the post-synaptic density (PSD) fraction of brain structures in rats following cocaine abstinence in an enriched environment, in an isolated condition, with extinction training or without instrumental task.

| **GluN1** | Yoked saline | Yoked cocaine | Cocaine self-administration | Statistical analysis |
| --- | --- | --- | --- | --- |
| **Cocaine abstinence in an enriched environment (n=8 rats/group)** | | | | |
| **PLC** (H) | 100 ± 1.79 | 98.8 ± 2.33 | 98.4 ± 2.46 | F(2, 21)=0.14; p=0.870 |
| **PLC** (PSD) | 100 ± 6.19 | 97.7 ± 2.52 | 90.5 ± 3.01 | F(2, 21)=1.39; p=0.272 |
| **ILC** (H) | 100 ± 6.29 | 107.3 ± 5.39 | 98.3 ± 4.63 | F(2, 21)=0.75; p=0.484 |
| **ILC** (PSD) | 100 ± 5.28 | 110.2 ± 2.71 | 112.9 ± 7.22 | F(2, 21)=1.59; p=0.228 |
| **dHIP** (H) | 100 ± 6.71 | 103.2 ± 9.90 | 113.0 ± 10.35 | F(2, 21)=0.36; p=0.701 |
| **dHIP** (PSD) | 100 ± 5.75 | 107.5 ± 3.31 | 101.8 ± 3.31 | F(2, 21)=0.85; p=0.442 |
| **vHIP** (H) | 100 ± 4.38 | 99.1 ± 4.54 | 98.5 ± 4.37 | F(2, 21)=0.03; p=0.972 |
| **vHIP** (PSD) | 100 ± 4.57 | 103.7 ± 4.32 | 102.0 ± 3.80 | F(2, 21)=0.19; p=0.828 |
| **dSTR** (H) | 100 ± 11.23 | 95.3 ± 9.56 | 104.9 ± 7.34 | F(2, 21)=0.25; p=0.778 |
| **dSTR** (PSD) | 100 ± 4.26 | 103.5 ± 3.40 | 103.5 ± 3.60 | F(2, 21)=0.29; p=0.752 |
| **vSTR** (H) | 100 ± 4.58 | 108.1 ± 8.92 | 102.7 ± 5.26 | F(2, 21)=0.40; p=0.674 |
| **vSTR** (PSD) | 100 ± 2.78 | 104.0 ± 4.69 | 98.3 ± 1.93 | F(2, 21)=0.76; p=0.479 |
| **BLA** (H) | 100 ± 2.41 | 106.9 ± 4.49 | 107.1 ± 2.52 | F(2, 21)=1.51; p=0.243 |
| **BLA** (PSD) | 100 ± 2.01 | 99.3 ± 1.71 | 92.9 ± 3.57 | F(2, 21)=2.32; p=0.123 |
| **Cocaine abstinence in an isolated condition (n=7 rats/group)** | | | | |
| **PLC** (H) | 100 ± 1.32 | 101.0 ± 2.93 | 100.7 ± 4.46 | F(2, 18)=0.03; p=0.974 |
| **PLC** (PSD) | 100 ± 2.08 | 99.4 ± 2.36 | 100.5 ± 2.77 | F(2, 18)=0.05; p=0.950 |
| **ILC** (H) | 100 ± 13.23 | 96.9 ± 12.98 | 99.6 ± 4.98 | F(2, 18)=0.02; p=0.978 |
| **ILC** (PSD) | 100 ± 12.31 | 80.6 ± 6.20 | 88.9 ± 5.03 | F(2, 18)=1.32; p=0.292 |
| **dHIP** (H) | 100 ± 5.85 | 98.3 ± 5.50 | 93.4 ± 2.41 | F(2, 18)=0.51; p=0.610 |
| **dHIP** (PSD) | 100 ± 5.30 | 102.1 ± 5.20 | 102.1 ± 4.88 | F(2, 18)=0.06; p=0.946 |
| **vHIP** (H) | 100 ± 9.32 | 109.9 ± 5.96 | 102.8 ± 7.50 | F(2, 18)=0.44; p=0.653 |
| **vHIP** (PSD) | 100 ± 9.29 | 91.3 ± 5.22 | 88.3 ± 6.47 | F(2, 18)=0.72; p=0.502 |
| **dSTR** (H) | 100 ± 9.33 | 99.6 ± 10.99 | 110.2 ± 16.05 | F(2, 18)=0.23; p=0.796 |
| **dSTR** (PSD) | 100 ± 5.87 | 90.5 ± 4.79 | 86.9 ± 5.76 | F(2, 18)=1.51; p=0.248 |
| **vSTR** (H) | 100 ± 4.68 | 111.2 ± 15.15 | 109.4 ± 7.76 | F(2, 18)=0.35; p=0.710 |
| **vSTR** (PSD) | 100 ± 3.83 | 101.0 ± 7.66 | 109.9 ± 5.46 | F(2, 18)=0.87; p=0.436 |
| **BLA** (H) | 100 ± 6.60 | 105.3 ± 15.38 | 130.5 ± 9.59 | F(2, 18)=2.14; p=0.147 |
| **BLA** (PSD) | 100 ± 10.38 | 95.4 ± 15.31 | 101.9 ± 4.13 | F(2, 18)=0.09; p=0.912 |
| **Cocaine abstinence with extinction training (n=8 rats/group)** | | | | |
| **PLC** (H) | 100 ± 7.86 | 98.3 ± 9.29 | 97.4 ± 7.33 | F(2, 21)=0.03; p=0.975 |
| **PLC** (PSD) | 100 ± 4.90 | 97.9 ± 14.09 | 103.6 ± 7.87 | F(2, 21)=0.09; p=0.915 |
| **ILC** (H) | 100 ± 2.45 | 102.3 ± 3.93 | 103.7 ± 3.34 | F(2, 21)=0.33; p=0.725 |
| **ILC** (PSD) | 100 ± 2.78 | 99.4 ± 4.01 | 99.5 ± 9.00 | F(2, 21)=0.01; p=0.997 |
| **dHIP** (H) | 100 ± 7.62 | 90.3 ± 8.30 | 90.7 ± 4.32 | F(2, 21)=0.62; p=0.549 |
| **dHIP** (PSD) | 100 ± 3.37 | 108.6 ± 5.66 | 113.0 ± 4.14 | F(2, 21)=2.15; p=0.141 |
| **vHIP** (H) | 100 ± 2.50 | 104.4 ± 9.06 | 107.1 ± 7.34 | F(2, 21)=0.27; p=0.766 |
| **vHIP** (PSD) | 100 ± 3.01 | 101.7 ± 6.12 | 103.7 ± 6.25 | F(2, 21)=0.12; p=0.890 |
| **dSTR** (H) | 100 ± 3.03 | 108.5 ± 5.48 | 114.2 ± 8.43 | F(2, 21)=1.38; p=0.273 |
| **dSTR** (PSD) | 100 ± 2.60 | 95.1 ± 4.88 | 92.0 ± 4.41 | F(2, 21)=0.98; p=0.393 |
| **vSTR** (H) | 100 ± 2.59 | 104.6 ± 7.69 | 101.8 ± 5.56 | F(2, 21)=0.17; p=0.846 |
| **vSTR** (PSD) | 100 ± 2.61 | 97.9 ± 3.04 | 95.5 ± 2.36 | F(2, 21)=0.72; p=0.499 |
| **BLA** (H) | 100 ± 0.80 | 99.7 ± 1.92 | 102.1 ± 1.55 | F(2, 21)=0.73; p=0.492 |
| **BLA** (PSD) | 100 ± 0.86 | 99.8 ± 0.92 | 99.4 ± 2.10 | F(2, 21)=0.04; p=0.961 |
| **Cocaine abstinence without instrumental task (n=8 rats/group)** | | | | |
| **PLC** (H) | 100 ± 5.16 | 104.4 ± 6.05 | 103.1 ± 5.25 | F(2, 21)=0.17; p=0.844 |
| **PLC** (PSD) | 100 ± 4.44 | 89.2 ± 6.63 | 97.2 ± 6.13 | F(2, 21)=0.92; p=0.413 |
| **ILC** (H) | 100 ± 5.02 | 103.5 ± 4.69 | 100.6 ± 4.27 | F(2, 21)=0.16; p=0.856 |
| **ILC** (PSD) | 100 ± 2.23 | 99.6 ± 2.23 | 99.3 ± 4.16 | F(2, 21)=0.01; p=0.988 |
| **dHIP** (H) | 100 ± 3.09 | 99.7 ± 7.10 | 86.9 ± 7.76 | F(2, 21)=1.41; p=0.267 |
| **dHIP** (PSD) | 100 ± 1.02 | 97.8 ± 1.34 | 99.6 ± 2.12 | F(2, 21)=0.55; p=0.582 |
| **vHIP** (H) | 100 ± 4.77 | 97.5 ± 6.54 | 96.7 ± 7.22 | F(2, 21)=0.08; p=0.927 |
| **vHIP** (PSD) | 100 ± 5.27 | 98.7 ± 5.10 | 96.5 ± 3.85 | F(2, 21)=0.14; p=0.873 |
| **dSTR** (H) | 100 ± 11.29 | 95.5 ± 9.68 | 95.3 ± 5.90 | F(2, 21)=0.08; p=0.921 |
| **dSTR** (PSD) | 100 ± 8.96 | 88.1 ± 7.04 | 97.5 ± 11.11 | F(2, 21)=0.46; p=0.635 |
| **vSTR** (H) | 100 ± 6.50 | 98.3 ± 2.81 | 99.1 ± 6.15 | F(2, 21)=0.02; p=0.976 |
| **vSTR** (PSD) | 100 ± 2.97 | 103.1 ± 4.23 | 113.2 ± 4.42 | F(2, 21)=2.98; p=0.073 |
| **BLA** (H) | 100 ± 5.27 | 113.8 ± 5.66 | 104.9 ± 3.17 | F(2, 21)=2.11; p=0.146 |
| **BLA** (PSD) | 100 ± 9.69 | 99.7 ± 12.40 | 84.9 ± 10.62 | F(2, 21)=0.62; p=0.548 |

ILC- infralimbic cortex, PLC- prelimbic cortex, dHIP- dorsal hippocampus, vHIP- ventral hippocampus, dSTR- dorsal striatum, vSTR- ventral striatum, BLA- basolateral amygdala, H- homogenate, PSD- postsynaptic density. All data are expressed as mean ± SEM [% of control]. N= 7-8 rats/group.

**Fig. S1** Changes in the expression of GluN2A subunit in the whole homogenate of brain structures in rats following cocaine abstinence A) in an enriched environment; B) in an isolated condition; C) with extinction training; D) without the instrumental task. ILC- infralimbic cortex, PLC- prelimbic cortex, dHIP- dorsal hippocampus, vHIP- ventral hippocampus, dSTR- dorsal striatum, vSTR- ventral striatum, BLA- basolateral amygdala. All data are expressed as mean ± SEM. N= 7-8 rats/group.

A) B)

C) D)

**Fig. S2** Changes in the expression of GluN2A subunit in the PSD fraction of brain structures in rats following cocaine abstinence A) in an enriched environment; B) in an isolated condition; C) with extinction training; D) without the instrumental task. ILC- infralimbic cortex, PLC- prelimbic cortex, dHIP- dorsal hippocampus, vHIP- ventral hippocampus, dSTR- dorsal striatum, vSTR- ventral striatum, BLA- basolateral amygdala, PSD- postsynaptic density. All data are expressed as mean ± SEM. N= 7-8 rats/group.

A) B)

C) D)
